# The Sciences During the New Common: A Missed Opportunity?

**DOI:** 10.1007/978-3-030-65355-2_16

**Published:** 2021-03-20

**Authors:** Maurits Kaptein

**Affiliations:** 1 Tilburg University, Tilburg, The Netherlands; 2 Tilburg University, Tilburg, The Netherlands; 3 Tilburg University, Tilburg, The Netherlands; 4 Tilburg University, Tilburg, The Netherlands; Jheronimus Academy of Data Science, Tilburg University, Tilburg, The Netherlands

## Abstract

By Wednesday, July 22, 2020, the coronavirus had killed over 611,000 people and infected over fourteen million globally. It devastated lives and will continue to do so for a long time to come; the economic consequences of the pandemic are only just starting to materialize. This makes it a challenging time to write about the new common. However, we need to start somewhere. At some point, we need to reflect on our own roles, the roles of our institutions, the importance of our economy, and the future fabric of everyday life. In this chapter, I will discuss one minor—and compared to the current crisis seemingly inconsequential—aspect of the new common: I will discuss my worry that we are on the verge of missing the opportunity to properly (re-)define the role of the sciences as we move from our old to our new common.

By Wednesday, July 22, 2020, the coronavirus had killed over 611,000 people and infected over 14 million globally (World Health Organization 2020). It devastated lives and will continue to do so for a long time to come; the economic consequences of the pandemic are only just starting to materialize. This makes it a challenging time to write about the new common. However, we need to start somewhere. At some point, we need to reflect on our own roles, the roles of our institutions, the importance of our economy, and the future fabric of everyday life. Here I will discuss one minor—and compared to the current crisis seemingly inconsequential—aspect of the new common: I will discuss my worry that we are on the verge of missing the opportunity to properly (re-)define the role of the sciences as we move from our old to our new common.

## What Is Science?

Many have discussed the role of the sciences during and after the current crisis. Most scientists have been positive: the crisis has highlighted the importance of the sciences for policymaking, and the general audience is more and more relying on science to develop new solutions (Nature Methods Editorial Team 2020). However, despite this, I personally fear the current crisis is reinforcing a narrow view of the sciences that will hinder the scientific community, and our society, in going forward. To clarify, I need a definition of the sciences, or simply an answer to the question “What is science?” This is admittedly tricky. However, luckily, some of the giants of the sciences—Richard Feynman in this case—have provided an answer (Feynman 2009):

The word is usually used to mean one of three things… Science means, sometimes, a special method of finding things out. Sometimes it means the body of knowledge arising from the things found out. It may also mean the new things you can do when you have found something out.

Starting from the final meaning, yes, the current crisis has amplified our collective belief in the use of the sciences relating to the “new things we can do.” Our confidence that we will be able to develop a vaccine demonstrates this point (Yamey et al. 2020). Regarding the second meaning, the crisis has also amplified the role of the sciences in relationship to the knowledge it creates. Policymakers have en masse turned to the sciences for answers. How quickly will the virus spread? How can we stop the spread of the virus? How will the spread of the virus be affected if we open up our schools? All of these are questions that policymakers have asked scientists to answer (see, e.g., Gatto et al. 2020).

However, the first, and foremost, meaning of the word science as identified by Feynman seems not to have been reinforced during the current crises and, consequently, risks losing importance in the new common. As people turn to the sciences for answers and solutions, we run the risk of losing its value as a *special method of finding things out*.

Omission of this first-mentioned role of the sciences (a) poses a threat to the societal confidence in the sciences and (b) hinders the efficiency by which we find answers to important problems. The first consequence is easily motivated: if we pretend science has the correct answer to every question—whereas in reality it only has uncertain answers or none at all—we easily damage the confidence of society in the sciences. Currently, this scenario seems to be unfolding in the case of hydroxychloroquine (Mehra et al. 2020, now retracted). As the answers science is providing are understandably mixed, regretfully, the heated debate regarding its efficacy leaves the general population with a diminished trust in the sciences as a whole.

The second consequence is less well understood or appreciated, even by some scientists. If we pretend the sciences have direct access to the truth, we ignore a large part of the sciences that has focused on finding out these truths to begin with. The honest answer scientists should give to many questions should be, “we don’t know, but we do know how to find out.” Such honesty will not only prevent a diminished trust in the sciences, it would also speed up and improve our decision-making. Allow me to illustrate.

## A Special Method of Finding Things Out

A recently fiercely debated question was “How will the spread of the virus be affected if we open up our schools?” We have settled for opening up our schools as the answer provided by the sciences was that the spread will only be affected in a limited way. The honest answer, however, is highly uncertain, as was, in this case, understood even by a large group of schoolteachers who argued against “being experimented upon.” These teachers understandably did not want to be part of the informal trial of finding out the effects of opening up our schools (see, Kuiper 2020).

I say understandably here because I would side with the school teachers that they do not want to be part of an experiment. Or, more precisely, they should not want to be part of the current poorly designed experiment. The current experiment is poorly designed because we are opening up the schools in the whole country in one go, and we are simultaneously changing a myriad of other policies. Hence, no matter the outcome, we will never truly learn anything about the effects of opening up the schools as the only variable. Any observed effect could easily be attributed to other policy changes (or even simply to the passing of time). We are not really finding things out.

If we had not ignored our special methods of finding things out, we would have acted differently (see, e.g., Robbins 1952). We could easily have set up a much more controlled experiment in which some schools opened up and some did not: an experiment in which we carefully sampled these schools, and carefully administered the “treatment”; an experiment in which we carefully designed the outcome measure and monitored the effect of our intervention; by all means, simply an experiment that did not willfully ignore the “special method of finding things out.”

## The Societal Value of Efficiently Finding Things Out

It is often argued that people do not want to be part of the experiment that I just described simply because they do not want to be experimented upon (ter Weijden 2020). People turn to the sciences for answers and facts, not for “ways of finding things out.” However, I think this is true only because scientists fail to properly explain the value of efficiently finding things out, which, in the case of the COVID-19 policy, seems easy enough to do as, in reality, it is not just the opening of schools that we are poorly experimenting with. We are carrying out the same poorly designed experiments with a myriad of other policies. Conversely, a well-designed experiment would be greatly beneficial for all involved (see, e.g., Eckles and Kaptein 2019).

Assuming we all want to “open up” as much as possible, and further assuming that each policy change takes 1 month before its effects are known, we are currently on the following path:

- Month 1: Total lockdown. The spread halts.
- Month 2: Make 6 policy changes that ease the restrictions simultaneously. The spread seems not to pick up.
- (Hypothetical) Month 3: Make 6 additional policy changes. The spread picks up again.
- (Hypothetical) Month 4: Reverse the last 6 changes. The spread decreases again.
- (Hypothetical) Month 5: re-instantiate 4 policies. The spread does not pick up.
- (Hypothetical) Month 6 and the beginning of winter: The spread picks up, and we have no clue which policy measure had which effect. Let us go to lockdown again.

In the end, we will have spent months in semi-lockdowns, having learned very little. Alternatively, we could have been on this path:

- Month 1: Total lockdown. The spread halts.
- Month 2: We carry out carefully designed experiments with varying policies in multiple, mutually comparable regions in the Netherlands (or Europe). We end up with proper estimates of the effects of each and every policy.
- (Hypothetical) Month 3: We roll out the optimal policy according to our estimates.

This latter process saves months and brings us valuable knowledge. It safely opens up our society faster, at smaller costs. And, by the time winter hits, we would know exactly which policies to re-instate.^1^

I have no doubt that, given our ability to convince our whole society to stay indoors for months, we should be able to communicate the societal benefits of carrying out carefully designed experiments. This is especially true if we had not sold our current poorly designed experiment as the “the best decision based on the facts,” but if we had initiated the general public into this all too often overlooked use of the sciences: *that of finding things out*.

## Footnotes

1 Regretfully, we now (early Nov. 2020) seem to be, in the Netherlands, nearing month 4 of the hypothetical scenario above: we are on the verge of rolling back a number of restrictions as our COVID-19 case numbers are increasing and we do not really know which exact policy change has caused the uptake.
